# Sentinel lymph node biopsy (SLNB) vs. axillary lymph node dissection (ALND) in the current surgical treatment of early stage breast cancer

**Published:** 2015

**Authors:** M Gherghe, C Bordea, A Blidaru

**Affiliations:** * Department of Nuclear Medicine, “Prof. Dr. Al. Trestioreanu” Institute of Oncology, Bucharest, Romania; ** “Carol Davila” University of Medicine and Pharmacy, Bucharest, Romania; Department of Oncologic Surgery II, “Prof. Dr. Al. Trestioreanu” Institute of Oncology, Bucharest, Romania

**Keywords:** breast cancer, sentinel lymph node biopsy (SLNB), axillary lymph node dissection (ALND)

## Abstract

The identification and biopsy of the sentinel lymph node has become a standard method of treatment for stage I and II breast cancer in the last decades, taking into account the fact that the management of the axilla in patients with breast cancer has evolved from the routine lymphadenectomy to a selective attitude, based on the histopathological evaluation of the sentinel lymph node, as well as on the tumor and on the patients’ characteristics.

Since the introduction of the method into clinical practice, in 1994, different methods of identification have been used (radioisotope injection, vital blue dye, or, more recently, lipophilic contrast agent for ultrasound visualization or paramagnetic nanoparticles (NPs) or the method of indocyanine green fluorescence), each presenting certain limits, but the radioisotopic method proving the most accurate. Moreover, during the development of the method, beside the standard indications specific for T1 or T2 breast tumor, without clinical or imagistic axillary adenopathies, their extension to a series of other particular situations such as the following, has been tried: ductal carcinoma in situ, multicentre tumors, after excisional biopsy or tumors preoperatively treated by neoadjuvant chemotherapy.

The aim of the paper is to present the progress made regarding the current stage in the use of sentinel lymph node technique in breast cancer, while mentioning the established indications, as well as the ones that are still debating and need further studies. Likewise, the cases in which the axillary lymph node dissection remains the major indication for treatment of the axilla, in patients with early stage breast cancer, will be discussed.

## Introduction

Breast cancer represents the most frequent form of cancer in women worldwide, respectively 30% of all the cancer types in women from European countries [**1**]. The incidence of breast cancer in the European Union, in 2006, was of 110,3/ 100000 women, and the annual mortality rate due to breast cancer was of 25/ 100000 women [**2**].

The rising incidence is mostly due to the introduction of the screening programs, which allow the detection of infraclinical breast tumors. However, in Romania, over 60% of breast cancers [**3**] are detected in advanced stages of the disease, stage III or IV, while the patient is hospitalized, due to the non-application of screening methods as well as the insufficient medical education of the population. 

The prognosis of the patients with breast cancer depends on many factors: age of the patient at the moment of diagnosis, characteristics of the tumor – especially its dimension, histological malignancy grading and the hormone receptors status, the presence of metastases in the axillary lymph nodes [**4**]. However, among all of these, the presence and the extension of the axillary lymph nodes involvement, represents an important prognosis factor, with major impact on further therapeutic decision. It was demonstrated that the presence of the metastases in the axillary lymph node drainage system decreases the 5-year survival rate, with approximately 28-40% [**5**]. 

The standard treatment of breast cancer consists of a radical mastectomy with the dissection of the axillary lymph nodes. This principle of radical resection has been considered the standard surgical treatment for a long time, because it was thought that the dissemination of tumor cells is firstly produced locally, in the regional lymph nodes and then distantly. As a result, an aggressive local control could have prevented the distant metastasis and would have decreased the mortality rate due to breast cancer. 

However, the hypothesis elaborated by Fisher et al., affirmed that in breast cancer, the systemic dissemination is precociously produced during the disease, but its manifestations could remain clinically silent for a long time [**6**]. That is why an aggressive local control would not determine a higher survival rate. This hypothesis was the first step in the development of a conservative approach in breast cancer, the studies coordinated by Fisher and Veronesi demonstrating that the limited resection followed by radiotherapy determines a long-term survival rate equivalent with that one obtained after the classical mastectomy [**7**]. 

Therefore, in the last decades, the surgical conservative treatment, has consisted of limited breast resection and the sentinel lymph node dissection, starting to be applied more often and having similar results regarding the survival rate, but also registering a significant decrease of postoperative complications (upper limb lymphedema, pain, sensitivity disorders) and a less dramatic psychological impact on breast cancer patients. 

**Sentinel lymph node concept in breast cancer**

The sentinel lymph node procedure was described for the first time in parathyroid cancer by Gould et al., in 1960, and in penile cancer by Cabanas, in 1977. However, in 1992, the procedure was clinically applied by using vital blue dye and radioisotopes. In 1994, Armando Giuliano described SLND as a safe procedure for staging the axilla, and later on, Umberto Veronesi confirmed the utility of the method by performing clinical trials. 

The hypothesis proposed by Cabanas claimed that the migration of the tumor cells is done through a lymphatic channel to only one lymph node before other nodes from the same lymphatic drainage basin [**8**]. In this way, the sentinel lymph node is the first node that receives a lymphatic drainage directly from the primary tumor, and, its histopathological examination could predict the status of the other nodes.

In Romania, the sentinel lymph node biopsy technique in the surgical treatment of breast cancer was introduced in 2003, in “Prof. Dr. Al. Trestioreanu” Institute of Oncology Bucharest, by the group coordinated by Prof. Dr. Al. Blidaru, who has done over 800 such procedures until present. This technique has become the standard method of staging the axilla in women with T1-T2 breast tumors, without clinical or imagistic signs of axillary adenopathies. 

By definition, the sentinel lymph node is the first node that receives direct lymphatic drainage from the tumor. During the use of the method, the concept of sentinel lymph node has had different definitions: the closest node to the primary tumor, the first node that can be seen on the lymphoscintigraphy, any radioactive node detected intraoperatively with the gamma probe or the node that fixes vital blue dye. All these definitions have proved incorrect, leading to that one mentioned above, which is unanimously accepted today.

**The radioisotopic technique of identifying the sentinel lymph node**

To identify the sentinel lymph node, lymphoscintigraphy uses different types of radiocolloids, labbeled with 99mTc, with dimensions of the particles ranging between 3 and 5000 nm. The 99mTc-nanocolloid is used in Romania and most of the European countries, being a compound with particles of 5-100 nm.

There is a general agreement that a radiocolloid with the majority of particles ranging between 100 and 200 nm in size can be considered the best compromise between fast lymphatic drainage and optimal retention in the sentinel lymph node [**9**]. 

No special preparation of the patient is necessary before performing the procedure. 

Pregnancy does not represent a contraindication for lymphoscintigraphy because it was proved that the radiation dose the fetus is exposed to, is very low, especially if doses lower than 10 MBq are used. For the breastfeeding women, a 24 hours withdrawal after the radiopharmaceuticals administration, is recommended.

In order to perform the procedure, a volume of 0,2-0,5 ml of nanocolloid is needed, with an activity of 5-20 MBq, which is usually injected peritumorally, followed by a gentle massage of the injection site. Intradermal, periareolar injections are frequently used and are especially indicated in the tumors localized in the central quadrant, as well as the subdermal injection above the tumor. During the development of the method, deep injection (intratumoral) has also been used, for which, there are not enough justifying arguments at present [**10**].

The images acquisition is obtained at 15 minutes after the injection and, afterwards, at 30 minutes interval until the visualization of the sentinel lymph node. Usually, it can be seen in the first 2-3 hours, but, sometimes, visualizations at 16-18 hours may be necessary. In more than 20% of the cases, more than one sentinel lymph node can be seen. 3-5 min acquisitions by using a gamma camera ecquipped with a low energy high-resolution collimator (LEHR), can be acquired. After the lymphoscintigraphic identification of the sentinel lymph node, it is marked on the skin by using a punctiform source of 99mTc, usually having an anterior oblique incidence to guide the surgical incision. SPEC-CT scans are also recommended if more areas of lymphatic drainage are identified, especially in the internal mammary lymph nodes, prepectoral or retropectoral lymph nodes. 

The surgical identification of the sentinel lymph node by introducing the intraoperative gamma probe counting is acquired at 16-20 hours after the injection [**11**]. It is necessary to have a high enough sensitivity of the detector, to identify a sentinel lymph node with a low radioactivity and to discriminate against an intense source determined by the proximity of the injection site. The sentinel lymph nodes that were intraoperatively identified are sent to histopathological examination, and, according to the result, the procedure of axillary lymphadenectomy is performed or not. 

**Current indications of sentinel lymph node biopsy**

**1.A.** Early-stage breast cancer (T1-T2), without axillary lymph nodes metastases, clinically or imagistically evidenced.

It is already, a routine clinical attitude, established by the therapeutic guidelines published by the American Society of Clinical Oncology, in 2005, that the correct surgical approach of the axilla in patients with early-stage breast cancer is the sentinel lymph node biopsy (SLNB). If its result does not indicate the presence of tumor cells, then the surgical intervention will not be completed by axillary lymphadenectomy (ALND). 

This indication is confirmed by many randomized clinical trials which have been published during the last couple of years, and, which have prospectively investigated if axillary lymphadenectomy can be avoided in patients who do not present tumor cells in the sentinel lymph node biopsy. The following clinical trials should be mentioned: NSABP (National Surgical Adjuvant Breast and Bowel Project) B32 [**12**-**15**], ALMANAC (Axillary Lymphatic Mapping Against Nodal Axillary Clearance) [**16**-**18**], Sentinella/ Givom (Gruppo Interdisciplinare Veneto di Oncologia Mammaria) [**19**-**21**]. All these publications included the results regarding the recurrence risk, rate of detection of the sentinel lymph node, adverse events and general survival rate. None of the studies evidenced significant differences between the mortality rates registered by the two types of surgical approach. For example, the study with the highest number of subjects, NSABP B32, reported a survival rate of 91,8% at 8 years, for the sentinel lymph node biopsy followed by axillary lymphadenectomy, compared to 90,3% for the sentinel lymph node biopsy alone. The mortality rate was of 4% in both studies. Moreover, the locoregional recurrence rate was of 2% in both cases and the distant recurrence rate was similar. 

As far as the adverse effects of the two types of axillary surgical approaches are concerned, the studies mentioned, showed that both the lymphedema (the most important complication of lymphadenectomy), as well as the less serious or frequent ones, such as infection, seroma, sensorial or motor deficit, were the most frequent or the most important from the clinical point of view, in patients who underwent a lymphadenectomy, compared to the ones who underwent a sentinel lymph node biopsy; for example, the ALMANAC study reported a 1% frequency rate of the moderate or severe lymphedema in the case of SLNB, compared with a 2% rate in the ALND case [**17**]. 

**1.B.** Early-stage breast cancer with isolated tumor cells or micrometastases in one or two biopsied sentinel lymph nodes. 

In the last couple of years it has been demonstrated that the axillary conservative surgery indication, limited only to the dissection of the sentinel lymph node (not completed by an axillary lymphadenectomy, the way it was recommended in previous studies), can be extended in case there is only one or two sentinel metastatic lymph nodes, if a limited breast resection followed by the conventional irradiation of the whole breast with fractionate doses is performed in these patients. 

The studies evidencing that there is no statistically significant difference as far as mortality and survival rates are concerned, without any sign of disease between the two therapeutic attitudes (SLNB or SLNB+ALND), were IBCSG 23-01 [**22**] and ACOSOG (American College of Surgeons Oncology Group) Z0011 [**23**]. Moreover, the locoregional or distant recurrence rates were similar, 7% for ALND and 5% for SLNB without ALND [**25**]. 

**2.** SLNB controversial indications in specific clinical circumstances. 

A. Multicentric breast cancer. The sentinel lymph node biopsy can be indicated in patients who have multicentric breast cancer that can be operated. 

No significant differences were evidenced in the ALMANAC [**18**] study, as far as the axillary recurrence risk was concerned, in patients with small multifocal tumors, compared to the ones with unifocal tumors, but there was a higher percent of false negative results in the last case. 

B. Ductal carcinoma in situ. It is recommended for SLNB in case mastectomy is decided to be performed or when the DCIS area has more than 5 cm. 

Ductal carcinoma in situ supposed the proliferation of the ductal epithelium, without any penetration or invasion of the basal membrane, and, therefore, without the existence of the risk of lymphatic dissemination in the locoregional lymph nodes. However, the SLNB indication is justified because it was noticed that a significant amount of cases, diagnosed with DCIS by a minimally invasive biopsy (core-biopsy or vacuum assisted biopsy), have also proved to present an invasive component after the result of the surgical excision [**24**]. 

In case the patients choose the conservative surgery of the breast, SLNB is not indicated, being considered a second option, in case of the histopathological examination of the resected part also highlights the existence of an invasive component. However, from a clinical and imagistic point of view, SLNB is recommended in patients in whom a palpable mass or a large area of microcalcifications (of over 5 cm) is identified, to which there is a high probability of existence of an invasive component. 

C. The previous breast or axillary surgery. The sentinel lymph node biopsy can be performed in patients with operable breast cancer, who were diagnosed by excisional biopsy or had a history of breast or axillary surgeries due to a benign pathology.

In a study published by Celebioglu F et al. [**25**], the SLN detection rate for those having undergone prior surgery was 96%, versus 95%, and the false negative rate was 10% versus 5,6%. The second study, published by Heuts EM et al. [**26**], was a nonrandomized study that included two groups: one group had undergone prior excisional biopsies, and the comparison group had undergone diagnostic core biopsies. Overall accuracy and sensitivity was 100 % in the first group, and there were no false negatives results. 

D. Preoperative neoadjuvant chemotherapy. The sentinel lymph node biopsy can be performed in patients who underwent a preoperative chemotherapy treatment, although the accuracy of the method is reduced. 

SLNB can replace ALND in patients in whom preoperatory neoadjuvant chemotherapy had as a result the complete resolution of axillary adenopathies. The SLN identification rate is significantly more reduced (77,6%) in patients who received a chemotherapy treatment compared to the ones preoperatively untreated (97%) [**27**], but the false-negative results were present in only 5,6% of the cases, compared with 7,4%. However, there are not enough studies to evaluate the axillary locoregional recurrence in patients who were chosen for SLNB after they carried out a preoperatory chemotherapy treatment. That is why, in such cases, radiotherapy aimed to the postoperative axillary area is indicated, to reduce the risk of axillary recurrence. Nevertheless, SLNB is not recommended in patients with tumors that were initially staged, such as T4abc, to which a favorable answer was obtained (with the reduction of the tumor stage) after chemotherapy.

**Indications for axillary lymph node dissection**

**1.** Early-stage breast cancer with lymph nodes metastases revealed as a result of sentinel lymph node biopsy, to which radical mastectomy is performed. 

This indication was established after some clinical trials, among which, the most important is IBCSG 23-01 [**22**]. However, both this clinical trial and others, which has referred to this pathology, had a low number of participants and the results were discordant, further researches being necessary. 

**2.** Locally advanced T3-T4 breast tumors or the case of tumors that present an inflammatory component. 

There are no results of the studies that have been performed in the last couple of years, which can modify the classical indication of axillary lymphadenectomy in locally advanced cancer. The few existing data in the literature do not recommend SLNB in these patients, in whom, usually, preoperative neoadjuvant chemotherapy is indicated, a clinical situation which was previously approached.

## Conclusions

In the modern treatment of breast cancer, the sentinel lymph node biopsy indication is present in many clinical circumstances, instead of classical axillary lymphadenectomy. It presents the advantage of conservative surgery, which significantly decreases the rate of postoperative complications, offering the patients a better quality of life and reducing the costs of patients care after surgery.

The results of the studies published in the last 8-10 years, have allowed the enlargement of the spectrum of clinical cases in which SLNB is enough, such as the presence of micrometastases in one or two sentinel lymph nodes, in which, until recently, axillary lymphadenectomy was indicated to complete the surgery. Moreover, the application of the method in more advanced stages was tried, after neoadjuvant chemotherapy, but with some limitations, which needs further researches. The clinical circumstances, such as the ductal carcinoma in situ or the excisional biopsy performed before surgery, in which it was thought that the sentinel lymph node technique had no benefits, have their importance in the most recent practical guidelines. 

The sustained work for the improvement and extension of the indications specific for the method, proves the major impact of this technique has had in the axillary surgery. However, studies on larger groups of patients are still necessary in order to explain some indications that are still debating.
